# Thyme Oil Reduces Biofilm Formation and Impairs Virulence of *Xanthomonas oryzae*

**DOI:** 10.3389/fmicb.2017.01074

**Published:** 2017-06-13

**Authors:** Akanksha Singh, Rupali Gupta, Sudeep Tandon, Rakesh Pandey

**Affiliations:** ^1^Department of Microbial Technology and Nematology, Central Institute of Medicinal and Aromatic Plants, Council of Scientific and Industrial ResearchLucknow, India; ^2^Chemical Processing Department, Central Institute of Medicinal and Aromatic Plants, Council of Scientific and Industrial ResearchLucknow, India

**Keywords:** biofilm, exopolysaccharide, thyme oil, virulence factors, quorum sensing

## Abstract

*Xanthomonas oryzae* pv. *oryzae* (Xoo), a common bacterial plant pathogen regulates its virulence and biofilm formation attribute *via* a chemical method of communication. Disabling this mechanism offers a promising alternative to reduce the virulence and pathogencity of the microorganism. In this study, the effect of thyme (THY) oil on Quorum Sensing mediated synthesis of various virulence factors and biofilm formation was analyzed. Treatment of Xoo with 500 ppm THY oil displayed a significant diminution in swimming, swarming, exopolysaccharide and xanthomonadin secretion. However, no effect was observed on bacterial growth kinetics and metabolic activity of the cells. Results were further authenticated by RT-qPCR as significant reduction in *motA, motB*, and *flgE* genes was observed upon THY oil treatment. Similarly, the expression of some extracellular enzyme genes such as endoglucanase, xylanase, cellobiosidase, and polygalacturonase was also found to be significantly reduced. However, biochemical plate assays revealed insignificant effect of 500 ppm THY oil on secretion of protease, cellulase, and lipase enzymes. The *rpfF* gene known to play a crucial role in the virulence of the phytopathogenic bacteria was also significantly reduced in the THY oil treated Xoo cells. HPTLC analysis further revealed significant reduction in DSF and BDSF signaling molecules when Xoo cells were treated with 500 ppm THY oil. Disease reduction was observed in *in vitro* agar plate assay as lesion length was reduced in THY oil treated Xoo cells when compared with the alone treatment. GC–MS result revealed thymol as the active and major component of THY oil which showed potential binding with rpfF gene. Application of 75 μM thymol resulted in downregulation of *gumC, motA, estA*, virulence *acvB* and *pglA* along with *rpfF.* The other genes such as *cheD, flgA, cheY*, and *pilA*, were not found to be significantly affected. Overall, the results clearly indicated THY oil and its active component Thymol to be a potential candidate for the development of anti-virulence agent which in future when applied in combination with conventional bactericides might not only help in lowering the dose of bactericides but also be successful in curbing the disease progression in rice.

## Introduction

*Xanthomonas oryzae* pv. *oryzae* (Xoo), a Gram-negative bacterium, causes bacterial blight, one of the most disastrous diseases of rice know to cause an annual yield losses up to 60% (Niño-Liu et al., 2006). It enters in plant tissue either through wounds or hydathodes and travels to the xylem vessels where it actively multiplies resulting in blight disease on rice leaves. Alternatives for disease control and management strategies are very limited and mainly dependent on the resistant cultivars and usage of antibiotics and chemicals (Mansfield et al., 2012). Given that there are limited effective antibacterial compounds for controlling Xoo, search to develop cost effective and novel strategies that have minimal environmental impact is the need of hour. One such new wrinkle is the use of anti-virulence or anti-quorum sensing (QS) agents that do not kill the bacteria but restrains the production of disease triggering virulence factors (Tay and Yew, 2013).

Like other bacterial genera production of an array of virulence factors such as extracellular polysaccharides (EPS) and various enzymes is under the control of cell-to-cell conversation system (QS). Xoo is known to produce signaling molecules by an intricate regulatory procedure relying on the concentration of QS molecules (Karatan and Watnick, 2009). The process is customarily engaged in governing genes associated with biofilm maturation, motility, competence, and virulence factors production (Xu et al., 2006; Hegde et al., 2009). QS is reported to exist in different bacterial species, with an assortment of diverse signaling molecules like *N*-acylhomoserine lactones (AHLs), diffusible signal factors (DSF) family signals, oligopeptides and autoinducers-2 (AI-2) (Chen et al., 2002; Barrios et al., 2006; Boon et al., 2008; Deng et al., 2010; Monnet and Gardan, 2015).

Among the different signaling molecules, DSF family signals produced by an array of bacterial pathogens such as *Xylella fastidiosa, X. campestris pv*. *campestris* (Xcc), *Stenotrophomonas maltophilia* including Xoo (Deng et al., 2014; Ryan et al., 2015) permit sensing their fellow natives and thereby coordinate individual task to form resistant biofilm. Key players involved in Xoo regulating the QS pathway include DSF, an intermediate chain fatty acid ‘*cis*-11-methyl-2-dodecenoic acid’ and BDSF(*cis*-2-dodecenoic acid). Basically, coordination and control of QS in Xanthomonads is carried out by the *rpf* gene cluster (He et al., 2010; Ryan and Dow, 2011).

Among the various factors responsible for eliciting disease symptoms, biofilm and cell degrading enzymes play an important role. Multiple studies have reported that QS-deficient mutants formed thinner and more disorganized biofilms compared to the wild-types (Tomlin et al., 2005). Recently, a study by Li and Wang (2014) reported effective restrainement of citrus canker symptoms caused by *X. citri* subsp. *citri* on upon application of foliar-applied biofilm inhibitors D-leucine and 3-indolylacetonitrile (IAN). Similarly, several essential oils were also found to inhibit biofilm formation capacity of *Pseudomonas aeruginosa* and *Staphylococcus aureus* (Kavanaugh and Ribbeck, 2012). Keeping in view these findings, we hypothesized that *Thymus vulgaris* plant oil (THY oil) might provide effective control against blight disease by acting as QS antagonists. To test the hypothesis, the present investigation was thus carried out with the aim to identify the role of THY oil on virulence traits such as biofilm formation, extracellular enzymes production, signaling molecules and consequently its effect on disease development when inoculated on rice plants along with Xoo.

## Materials and Methods

### *Xanthomonas* Strain and Growth Conditions

The *X. oryzae* pv. *oryzae* (Xoo) strain AS29 was isolated from susceptible rice cultivar BLB Pusa Basmati 1 (India) and further identified using 16S-rDNA gene sequence. The identified strain was submitted to GenBank database (Accession Number KX010418) and routinely grown on PME (Peptone Malt Extract) (HiMedia, India) plates (0.5% peptone, 3% malt extract, 1.5% bacto agar) at 26 ± 2°C.

### Spectrophotometric Biofilm Assay

The protocol for measuring biofilm production was carried out following the method described by Dunger et al. (2014). Overnight grown XooAS29 culture was inoculated into 2 mL PME broth with 1:1000 dilution so that OD_600_ reached 0.6. To the 96-well U-plastic titre plate (SPL Life Sciences, Co., Ltd; South Korea), 1 μL culture was added to 99 μL PME broth in each well. For evaluating the consequence of THY oil, 100–1000 ppm were initially used to assess their effect on biofilm formation. The titre plate was incubated without shaking at 28°C for 10 h. The incubated cells were then stained with crystal violet (CV) dye for 20 min. The unbound dye was removed by rinsing twice with sterile distilled water (SDW). Finally, the well-bound dye was solubilized in 200 μL dimethyl sulfoxide (100% DMSO) and quantified spectrophotometrically by recording absorbance at 595 nm.

### Microscopic Analysis of Biofilm

Since, 500 ppm oil was most efficient in the aforementioned method; the outcome of this concentration on biofilm (microscopic visualization) in contrast to XooAS29 alone (control) was measured using polyvinyl chloride (PVC) assay (Krzyściak et al., 2014). The XooAS29 culture (OD_600_ = 0.6) was supplemented into 1 mL of PME broth consisting of 500 ppm THY oil for 14 h at 28°C. The PVC plate wells were washed twice with SDW to eliminate the buoyant planktonic cells after the incubation time. The bacterial cells adhering to the surface were stained with 0.5% CV and 0.2% methylene blue (MB) dyes, respectively and incubated for 5 min at room temperature. The surplus dye was eliminated by pipetting out and the plates were again kept for air drying at room temperature. The dried plates were further imaged at a magnification of 20X (Olympus BH2, Japan) under a light microscope. The XooAS29 culture was allowed to grow on the cover glass submerged in PME medium in the absence and presence of specific concentration of oil and incubated for 10–12 h. The biofilm formed were further stained with green fluorescent dye 20 mM SYTO-9 and observed under fluorescent microscope (Leica DMR epifluorescence microscope, Germany).

### Metabolic Activity and Growth Kinetics

In the metabolic activity assay, a stock solution of 0.002% (w/v) resazurin reagent (Sigma, United Kingdom; filter sterilized) was prepared (Mariscal et al., 2009) and stored at -20°C. XooAS29 culture was grown with and without 500 ppm THY oil in PME broth and incubated for 24 h. The culture was centrifuged and the cells were washed with saline. Individually XooAS29 (100 μL) was added to each well of 96 well titre plate followed by addition of 20 μL resazurin solution. The resazurin fluorescence activity (*λ*_ex_: 560 nm and *λ*_em_: 590 nm) was measured after 60 min of incubation at 37°C. To test the growth inhibitory effect of THY oil, XooAS29 was grown in PME broth with and without effective concentrations of THY oil (500 ppm) using a shaking incubator for 8–13 h at 25 ± 2°C and the absorbance was recorded at 600 nm in triplicate samples collected hourly. Xoo cells treated with oil served as treatment while cells grown in the absence of oil was taken as control.

### Swimming, Swarming, and Wetness Assay

XooAS29 inoculum was grown as mentioned above and prior to carrying out the assay the final density was adjusted to 1 × 10^8^ CFU mL^-1^. For conducting the motility assays, XooAS29 cells mixed with 500 ppm THY oil were gently inoculated in the center of the solidified PME agar plates. 1 μL suspension droplets were carefully placed in the center of soft swimming plates (3% malt extract, 0.5% peptone, 0.3% agar), and (2) swarm plates (3% malt extract, 0.5% peptone, 0.5% agar), and kept for incubation at 25 ± 2°C. The swimming and swarming traits were evaluated after 24 h by calculating the diameter of the bacterial zone. An equivalent quantity of only XooAS29 inoculum without oil in the respective media served as control.

The wetness assay of the colonies on swarm plates was calculated by the capillary-drop method as delineated by Wang et al. (2005). Capillary tubes of 0.5 mL capacity were mildly positioned on the treatment and control colonies for 30 s and the length of fluid entered was measured in millimeters. Xoo cells treated with oil served as treatment while cells grown in the absence of oil was taken as control. Five tubes were placed on different points on swarm plates for calculating the average and SD values.

### Quantification of Exopolysaccharides (EPS) and Xanthomonadin

For EPS, XooAS29 culture with and without 500 ppm THY oil was grown on PME media for 24 h at 28 ± 2°C. To detect the production of EPS from bacterial cells, the cells were scraped off the plates and resuspended in 15 mL SDW. Centrifugation at 15,000 × g for 12 min was done and the supernatant was collected for further analysis. The collected supernatant was muddled up with 1% (w/v) potassium chloride and double volume of 100% ethanol and further incubated at -20°C overnight. Again centrifugation was done and the precipitated EPS was left overnight for drying at 55°C before determining the total dry weight. Expression of the results was done relative to the cell density.

Measurement of xanthomonadin pigment was done as mentioned in the method illustrated by Wang et al. (2015). The XooAS29 cells collected by centrifuging 4 mL broth suspension with and without 500 ppm THY oil was mixed with 1 mL 100% methanol. The concoctions were further incubated in darkness for 10 min kept on rotating shaker followed by centrifugation at 12,000 × *g* for 8 min to collect the supernatant. The xanthomonadin pigment was estimated by measuring the absorbance at OD_445_ and the result was denoted relative to the cell density measured before the assay (OD_595_).

### Extracellular Various Enzymatic Assays

The fresh colony of XooAS29 strain was grown in 10 mL of PME liquid medium in presence and absence of 500 ppm THY oil at a starting OD_600_ of 0.05. After incubation for 24 h, the XooAS29 culture at an OD_600_ of 1.8 was centrifuged at 12,000 rpm for 12 min and the supernatant obtained was used for enzymatic plate assays (Yang and Tseng, 1988). The extracellular protease activity was measured by a radial diffusion assay in agar plates containing skimmed milk as substrate with 0.5% skimmed milk and 2% (wt/vol) agar. The media was poured in the plates and wells of 6 mm diameter were cut out with the help of a cork borer. The cell suspension (with or without THY oil) was applied in the well of the plates. Clearing zones around the colony due to the utilization of the substrate were measured after 24 h of incubation at 28 ± 2°C.

Extracellular cellulase activity was calculated as explained previously (Maki et al., 2011) using 2% CMC (Sigma-Aldrich) as substrate. Xylanase activity was also assayed on 1% agarose plates containing 0.5% RBB-Xylan and PME agar media (He et al., 2010). Positive xylanase activity was indicated by production of a white halo around the well in the plate.

For endoglucanase activity, XooAS29 culture with and without oil was pipetted into 6 mm diameter wells cut into CMC agar plates (1% agar, 0.125% CMC in 0.05 M potassium phosphate buffer, pH 6.0). The plates were incubated for 48 h at 28°C and then developed with Congo red dye (Barber et al., 1997). Extracellular lipase/esterase activity was assayed on PME media containing 0.01% CaCl_2_ and 1% Tween 80 (Rajeshwari et al., 2005). The white crystals surrounding colonies was measured in medium containing CaCl_2_ and Tween 80 indicating positive lipase activity. All the assays were repeated three times, independently, in triplicates.

### Expression Analysis by Real-Time RT-qPCR

For RNA isolation, single celled colony of XooAS29 with and without 500 ppm THY oil was transferred to 10 mL PME medium at 28 ± 2°C and sampled when the OD_595_ reached 2.0 (Xu et al., 2015). RNA was isolated by following the manufacturer’s instructions of Trizol (Invitrogen, Carlsbad, CA, United States) method and RNA purity was evaluated with a NanoDrop ND-1000 spectrophotometer (NanoDrop Technologies, Wilmington, DE, United States). Total RNA was used for synthesizing first-stranded cDNA using c-DNA verso reverse transcriptase (Thermo Scientific) following the instructions mentioned in the kit. The realtime RT-PCR was carried out in 96-well plates using a 7500 fast real-time PCR system (Applied Biosystems) with a Sybr Green master mix (Fermentas) following the manufacturer’s instructions (Li and Wang, 2014). The gene expression was done in triplicate in a total volume of 25 μL including a passive reference dye (ROX) (Fermentas). The designing of gene specific primers (**Table 1**) was done on the basis of the available genome sequence of *X. oryzae* pv. oryzae strain KACC10331. The primers synthesized targeted 21 genes that were earlier recognized to be related to motility, chemotaxis, hydrolytic enzymes, virulence, and DSFs. As an endogenous control DNA gyrase subunit B encoding gene gyrB was used and the relative transcript level was calculated using the method 2^-deltadelta^ ^CT^ described by Livak and Schmittgen, 2001.

**Table 1:** Genes used in quantitative reverse transcription polymerase chain reaction.

Serial number	I.D. number/gene	Protein function	Primer (real time)
1	XOO3618	Xylanase	F-CAGACCTATCAGACGGTGCG R-GAGCTGGATGGGTCGATACG
2	XOO3300	Extracellular protease	F-CGACCGACTTCACCATCCTG R-CTTCATCAGGCTACCGTCGG
3	XOO4028	Endoglucanase	F-TGGTGTCGTTGGTGTCGTAG R-GGTTCTTCTGCAGCCAAGC
4	XOO4036	Cellulase	F-CCGTCTATGTCACCGACCTG R-CGAGCTTGACCACCGGATAG
5	XOO4035	1,4-Beta cellobiosidase	F-GAACGGGCATCCATCGAGAG R-GACATGACTCTGGGCCGATG
6	XOO4059	Lytic enzyme	F-CCGAGCCTGATCTCCAACGA R-GTCGGGGCAGGTTGATTCAG
7	XOO2738 (acvB)	Virulence protein	F-ATTGCGCGCCGAACATG R-CCCTGGATCCTTGTTACCGG
8	XOO2858 (cheD)	Chemotaxis protein	F-GTATCTGGTGGTGGACGACG R-CGAAGACCTTGGCCTCGATG
9	XOO2835 (cheY)	Chemotaxis protein (response regulator)	F-GTCAAGAATCTGCTGGGCGA R-GTCACCATCATCACCGGCA
10	XOO3370 (estA)	Lipase, esterase	F-TACACCGAGAGCAACGACAC R-CCCTCCTTGAACTCGTGGTC
11	XOO2567 (flgA)	Flagellar protein	F-CAGTCTGTCGATTCCATCCG R-CGCCACTTCCACCGTATTG
12	XOO2572 (flgE)	Flagellar biosynthesis hook protein	F-CGTCCAACAACATCGCCAAC R-GGATCGATATTGCCCTGCGA
13	XOO2830 (motA)	Mot A protein	F-CAGCGTATTGAAAGGTGCCG R-GATGTTGCTCCACTCGACGA
14	XOO2831 (motB)	Mot B protein	F-ACCACCTTGTCCACGCTTG R-ATAGCCCACCATCGCCAATC
15	XOO3178 (gumC)	gumC	F-GTCGGCAAAGATCGCCAAC R-TCTGGGCAGGCAAAGAAGG
16	XOO2699 (pglA)	Polygalacturonase	F-CGAGTTTCAGCGGCATCAC R-TTCCACCTTCCAGCACCAC
17	XOO0581 (pilA)	Fimbrial protein	F-TTCAAGCCACCCGAGCA R-CGCGGCAATTAGGTGGAAG
18	XOO2869 (rpfF)	Rpf F	F-CTCTTTCATGTGCCAGCGC R-GCGGATCACCTGTTCGACT
19	XOO2871 (*rpfG*)	Response regulator	F-CGTGGATCTGTTGCTGCTG R-AGTCGATCACACCCGCTTC
20	XOO2870 (*rpfC*)	Rpf C	F-ACGCGCGGACTGGATTA R-ACGCATGTCCATCGGCA
21	XOO0004 (gyrB)	DNA gyrase subunit B (Housekeeping gene)	F-ATGAACGCCGAGCAGCT R-AGCGCGTTGTCCTCGAT

### Quantification of QS Factors

XooAS29 culture with and without 500 ppm THY oil was individually allowed to grow as mentioned above and sampled when the OD_595_ reached 2.0. DSF (*cis*-11-methyl-2-dodecenoic acid) and BDSF (*cis*-2-dodecenoic acid) were quantified as illustrated by He et al. (2010) and Xu et al. (2015). Five liters of XooAS29 supernatant with and without 500 ppm THY oil was individually collected by centrifugation at 9,000 rpm for 40 min at 4°C. Equal volume of ethyl acetate was used for extracting the obtained supernatant whose pH was adjusted to 4.0. The removal of organic phase was done by rotary evaporator at 40°C, and the residue obtained was dissolved in 20 mL of methanol. The final dried extract obtained was dissolved in HPLC grade methanol. The signaling molecules present in ethyl acetate extracted samples were detected through prewashed aluminum HPTLC plates (10 cm × 10 cm), coated with silica gel 60- GF254 (Merck). 2 μl of each sample was applied in triplicate on preactivated TLC plate using the Camag Linomat 5 automated TLC applicator equipped with a 100-μl Hamilton syringe. Hexane: diethylether: acetic acid (8:2:0.1) was implemented as mobile phase to develop TLC plate in Camag twin glass chamber. After developing the TLC plate to 90 mm distance, the plate was removed and dried at room temperature for 1 h. Developed plate was documented under 254 and 366 nm, and then transferred into previously saturated iodine chamber to visualize the spot under day light. The plates were then scanned at 270 nm with a Camag TLC Scanner with winCATS 3 software, using the deuterium lamp. The densitograms was recorded and peak area of both samples was observed.

### Virulence Assay on Rice Plants

The leave samples were taken from 40- to 60-day-old susceptible rice cultivar BLB Pusa Basmati 1 plants grown in greenhouse conditions. The leaves were washed twice with SDW followed by clipping tips of leaves with sterile scissors dipped in saturated XOOAS29 (10^9^ cells/mL) grown with and without 500 ppm THY oil (Kauffman et al., 1973). As per the adopted method, approximately 10^6^ cells are expected to be deposited at the site of pathogen inoculation. The leaves were then placed on the water agar (0.5% (wt/vol) agar) plates and left for 3 days at 28°C. The lesion lengths were measured by comparing leaves clipped with scissors dipped in XooAS29 alone and with THY oil. The experiment was repeated three times in triplicates.

### Docking of Major Components of Oil with RpfF Protein

The crystal structure of the targeted protein, dsf synthase RpfF (PDB: 3M6M) was selected as receptor and retrieved from the RCSB protein data bank. The elimination of water molecules and selection of single peptide chain of receptor was performed using chimera software. All ligand structures were extracted from NCBI PubChem database in sdf format and converted into pdb format using online tool “Smile Converter.” The graphical user interface program “Auto-Dock Tool 4.2” was used in docking simulation. The ligand energy was also optimized by defining the rotable bond, torsion angle and submerging the non-polar hydrogen bonds. Autodock tool demands the precalculated grid-box which was set at 70, 70, and 70 A^0^ (x, y, and z) with 0.375 A^0^ grid spacing to include all the presented amino acid residues of ligand binding pocket of receptor. Lamarckian genetic algorithm method was applied to perform docking. The default settings were used for all the other parameters. Final docked conformations were clustured using a tolerance of 2 A^0^ RMSD and the docking log (dlg) files were analyzed. The best ligand-receptor structure from the docked structures was opted based on the binding energy and number of H-bonds formed between the target and ligand. The results were visualized using visualization tool “Discovery studio 4.5”.

### Effect of Thymol on XOOAS29 Biofilm Formation, Growth Kinetics, and Expression of Various Genes

After getting the best interaction with thymol and targeted protein, screening was done to find out the effective concentration of thymol (0, 10, 25, 50, 75, 100, 200, 300, 400, and 500 μM). The selection for the effective concentration was done on the basis of the concentration which showed no growth inhibitory effect on Xoo along with inhibitory outcome on the biofilm formation. The experiment was repeated three times in triplicates.

Also, RT-qPCR was performed to quantify and compare the levels of gene expression for *gumC, motA, estA*, virulence gene *acvB cheD, flgA, cheY, pilA, pglA* along with DSF related genes *rpfF, rpfC*, and *rpfG* in Xoo when exposed to thymol. qRT-PCR was repeated twice, with four independent biological replicates each time.

### Statistical Analysis

Each experiment was done in three technical replicates and three biological replicates. Mean significant values were determined by Student’s *t*-test using SPSS package (SPSS V16.0, SPSS, Inc., Chicago, IL, United States). Analysis of variance (ANOVA) followed by the post-test (Duncan’s Multiple Comparison Test) was also used to analyze significance between more than two treatments. ^∗^*P* < 0.05 and ^∗∗^*P* < 0.01 was considered statistically significant.

## Results

### THY Oil Suppresses XooAS29 Biofilm

**FIGURE 1:**
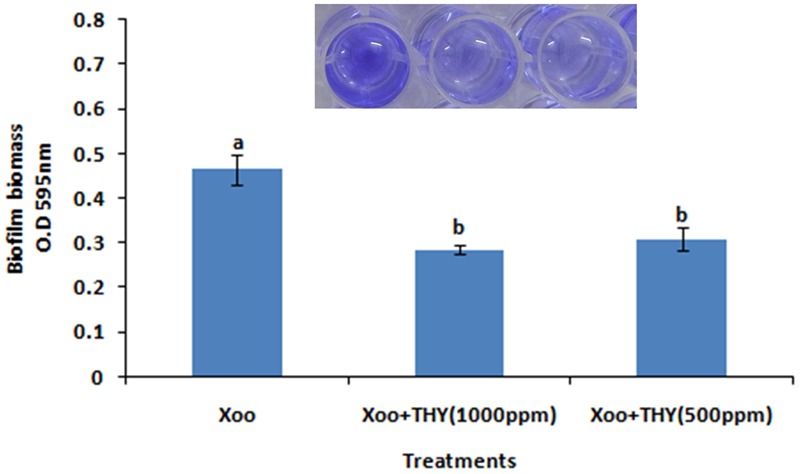
Different concentrations of THY oil suppresses biofilm of *X. oryzae* pv. *oryzae* (Xoo) strain AS29. Only “Xoo” served as control while “Xoo + THY (1000 ppm)” and “Xoo + THY (500 ppm)” represented the treatments used in the experiment. Results are means of three technical replicates and three biological replicates and error bar indicates the standard error. Different alphabets on vertical bars indicate significant dissimilarity among treatments (*P* < 0.05; Duncan’s multiple comparison test).

In the present study, it was attempted to probe whether biofilm formation was reduced in XOOAS29, when treated with THY oil or not. The Xoo strain AS29 (KX010418) was routinely used throughout the experiment. The result of micro titre plate based CV staining revealed a significant decrease in biofilm formation in a concentration dependent manner after 24 h of incubation at 28°C in THY oil treated XooAS29 cells. As shown in **Figure 1** and Supplementary Figure S1, THY oil at concentrations of 1000 and 500 ppm significantly reduced (1.64 and 1.50 fold respectively) biofilm forming ability of XooAS29.

**FIGURE 2:**
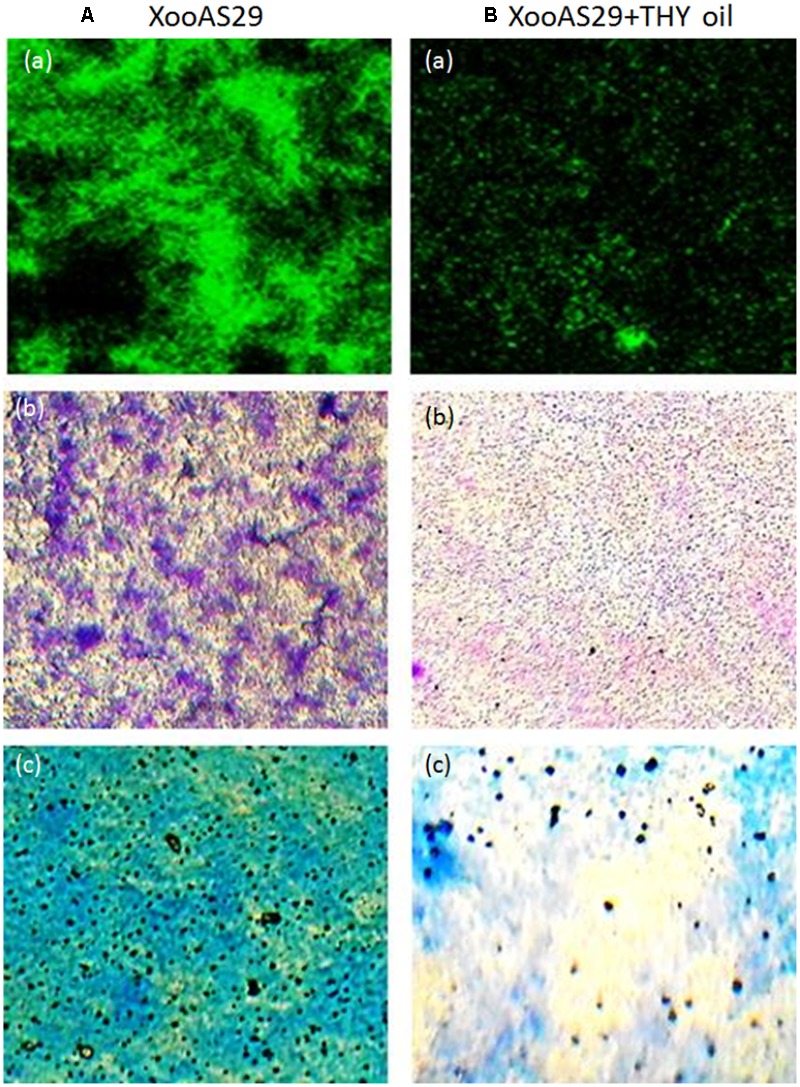
Microscopic observation of specific concentration of THY oil (500 ppm) to suppresses biofilm of *X. oryzae* pv. *oryzae* (Xoo) strain AS29. Xoo biofilm was grown in the absence or presence of 500 ppm of THY oil. Only “XooAS29” served as control while “XooAS29+ THY oil” represented the treatment in the figure. **(A,B)** Fluorescent microscope images; (a) SYTO-9-stained light microscope, (b) CV-stained light microscope images; and (c) MB-stained light microscope (20X, scale bar = 200 μm).

To further confirm the result obtained from quantitative assay microscopic visualization was done only for the lower concentration of THY oil (500 ppm) significantly inhibiting biofilm formation under light microscope. The images obtained from three different staining methods namely SYTO-9, CV and MB dye further reconfirmed the observation that biofilm production was significantly lessened when XooAS29 cells were treated with 500 ppm of THY oil than the alone Xoo cultures (control) (**Figure 2**).

### Effect of THY Oil on Metabolic Activity and Growth Kinetics of XooAS29

**FIGURE 3:**
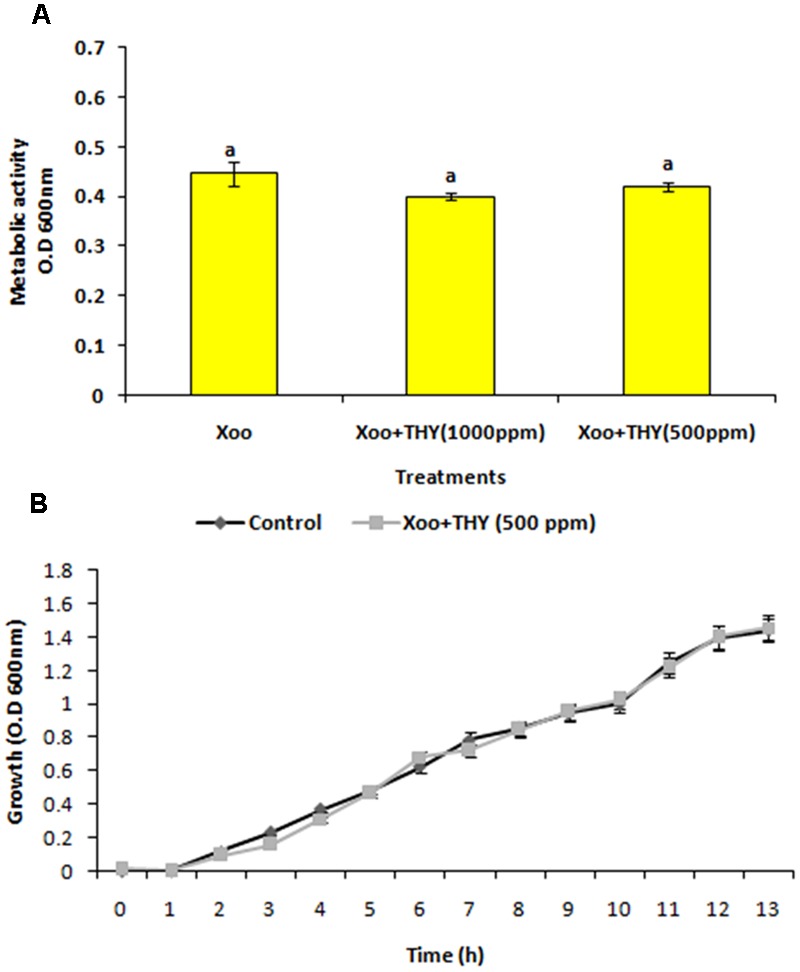
Effect of THY oil on **(A)** metabolic activity and **(B)** growth kinetics of *X. oryzae* pv. *oryzae* (Xoo) strain AS29. Only “Xoo” served as control while “Xoo+ THY (500 ppm)” and “Xoo+ THY (1000 ppm)” represented the treatments used in the experiment. Results are means of three technical replicates and three biological replicates and error bar indicates the standard error. Same alphabets on vertical bars indicate non-significant among treatments [*P* = 0.19 (Metabolic activity); Duncan’s multiple comparison test].

Since, the aim was to find an anti-virulence agent that only affected the secretion of virulence factors and did not kill the cells, the effect on XooAS29 on both metabolic activity and growth kinetics in the presence and absence of THY oil was investigated. The XooAS29 culture grown in the presence of both the 1000 and 500 ppm concentrations of THY oil had little/no significantly reduced Xoo metabolic activity (**Figure 3A**). Similarly, XooAS29 treated with THY oil (500 ppm) also showed no significant effect on growth of bacterial cells in comparison to the control set (**Figure 3B**).

### THY Oil Suppresses Swimming, Swarming, and Wetness Assay of XooAS29

**FIGURE 4:**
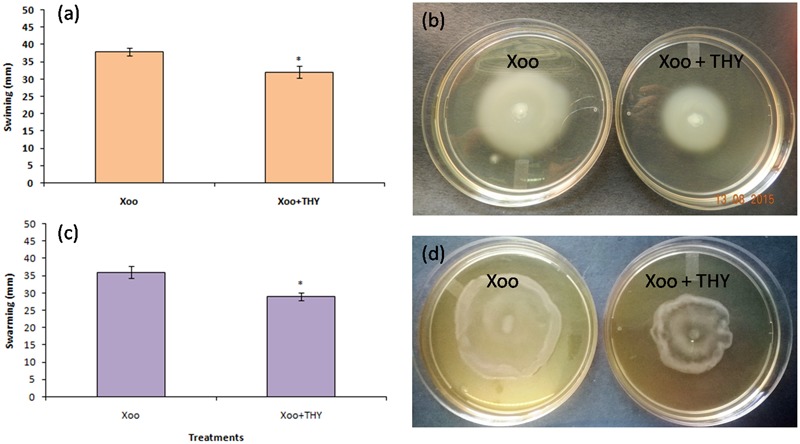
Effect of THY oil (500 ppm) on swimming and swarming of *X. oryzae* pv. *oryzae* (Xoo) strain AS29. For recording phenotypic variation in swimming **(a,b)** and swarming traits **(c,d)**, 1 μL of XooAS29 cells (1 × 10^8^ CFU mL^-1^) mixed with 500 ppm THY oil suspension and was carefully placed in the center of soft swimming plates (3% malt extract, 0.5% peptone, 0.3% agar), and on swarm plates (3% malt extract, 0.5% peptone, 0.5% agar). The plates were kept for incubation at 25 ± 2°C for 24 h. Xoo+THY in the figure depicted treatment while Xoo denoted control. Only “Xoo” served as control while “Xoo+ THY” represented the treatment in the figure. Results are means of three technical replicates and three biological replicates and error bar indicates the standard error. Asterisk indicates ^∗^*P* < 0.05.

Since, 500 ppm THY oil didn’t have any bactericidal effect on the XooAS29 cells along with significant reduction in biofilm formation; we tested the ability of XooAS29 to swim in low percentage agar medium in the presence of THY oil. After incubation at 28 ± 2°C for 24 h, Xoo AS29 cultured on plates with 500 ppm oil showed (*P <* 0.05) considerable reduction in swimming and swarming motility (1.19 and 1.24 fold, respectively) as compared to XooAS29 in the control plate. Small swimming and swarming diameters were observed in THY oil treatment which was in sharp contrast with the control sets where large diffused colony was observed on PME plates (**Figures 4a–d**).

In general, it is believed that bacterial cells need a certain amount of wetness for swarming movement on sticky surfaces. Thus, with the capillary-drop method, we evaluated the wetness of XooAS29 colonies grown on swarm plates with and without 500 ppm THY oil. As shown in Supplementary Figure S2, the wetness of the XooAS29 colony was significantly reduced (1.24 fold) in the presence of THY oil, suggesting that swarming defects by THY oil may be due to insufficient production of wetting agents or extracellular materials.

### THY Oil Reduces EPS Synthesis and Xanthomonadin Production of XooAS29

**FIGURE 5:**
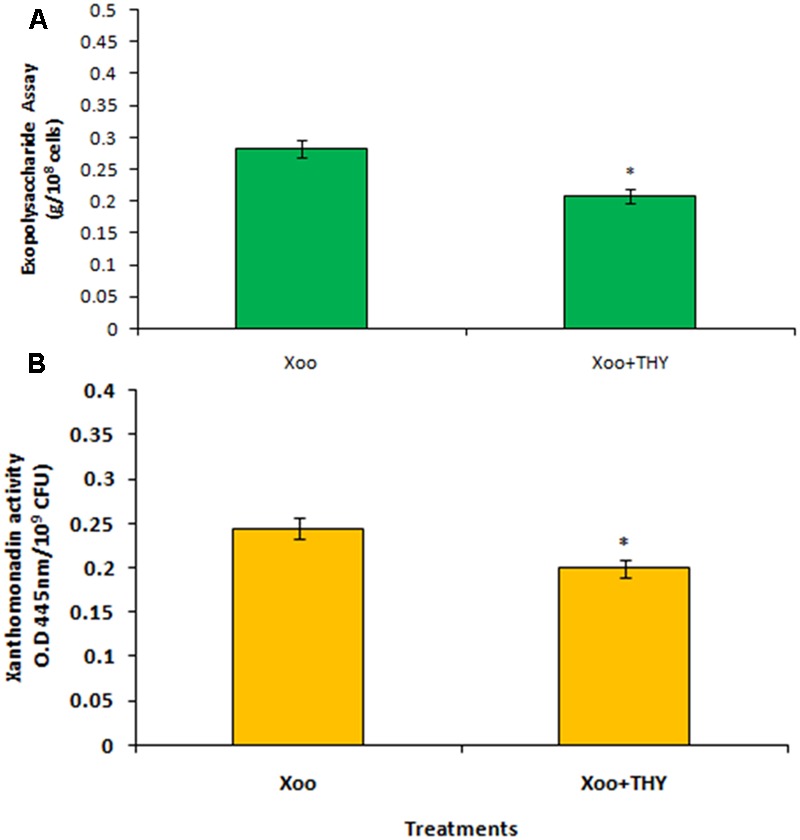
Specific concentration of THY oil (500 ppm) suppresses exopolysaccharide **(A)** and xanthomonadin activity **(B)** of *X. oryzae* pv. *oryzae* (Xoo) strain AS29. Only “Xoo” served as control while “Xoo+ THY” represented the treatment in the figure. Results are means of three technical replicates and three biological replicates and error bar indicates the standard error. Asterisk indicates ^∗^*P* < 0.05.

Production of EPS is a key determining factor of biofilm formation and is also correlated with the chemotactic movement in Xoo. Since the results revealed significant inhibition in biofilm formation of XooAS29 treated with 500 ppm of THY oil, we sought to extend our study by evaluating the effect of THY oil on EPS synthesis and xanthomonadin production by Xoo. The XooAS29 culture grown in the presence of 500 ppm THY oil significantly (*P <* 0.05) reduced (1.35 fold) EPS than the control treatment after 24 h of inoculation (**Figure 5A**). Interestingly, XooAS29 treated with 500 ppm THY oil also significantly (*P <* 0.05) reduced xanthomonadin which was about 1.23 fold less compared with the control set (**Figure 5B**).

### THY Oil Suppresses Extracellular Hydrolytic Enzymes Production of XooAS29

Several other virulence related phenotypes were assayed by assessing the extracellular enzyme production of XooAS29 both in the presence and absence of THY oil. As shown in **Table 2**, 500 ppm of THY oil was found to significantly reduce endogluconase and xylanase activity (1.55 and 1.94 fold, respectively). However, interestingly non-significant affect was observed in the case of protease, cellulase, and lipase activity as compared to control plate inoculated with only XooAS29.

**Table 2:** Effect of THY oil on different hydrolytic enzymes such as protease, cellulase, endogluconase, xylanase, and lipase of *X. oryzae* pv. *oryzae* (Xoo) strain AS29.

Extracellular hydrolytic	XooAS29	XooAS29 + 500 ppm
enzymes	(mm)	THY oil (mm)
Protease	22.00 ± 0.87	20.00 ± 0.29
Cellulase	29.00 ± 1.73	28.50 ± 1.15
Endogluconase	26.33 ± 1.28	17.00 ± 0.33^∗^
Xylanase	31.00 ± 1.15	16.00 ± 0.58^∗^
Lipase	14.00 ± 0.29	12.00 ± 1.44

*Results are means of three technical replicates and three biological replicates and error bar indicates the standard error. Asterisk indicates ^∗^*P* < 0.05.*

### Differential Gene Expression of XooAS29 with THY Oil

**FIGURE 6:**
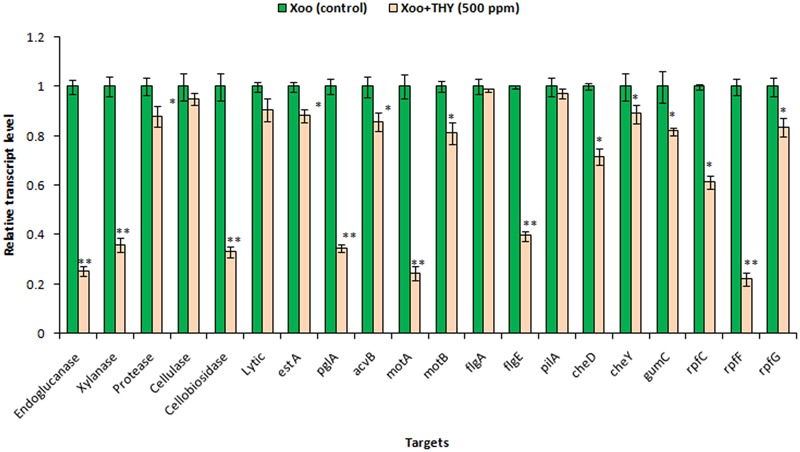
Differential gene expression in *X. oryzae* pv. *oryzae* (Xoo) strain AS29 in the absence and presence of THY oil (500 ppm) as revealed by quantitative real time polymerase chain reaction (qRT-PCR) analysis. Only “Xoo” served as control while “Xoo+ THY” represented the treatment in the figure. *gyrB* was used as an endogenous control. Results are means of three technical replicates and three biological replicates and error bar indicates the standard error. Asterisk indicates ^∗∗^*P* < 0.01 and ^∗^*P* < 0.05.

Previously, we showed that 500 ppm THY oil showed inhibitory effect on biofilm formation, EPS, xanthomonadin, and some of the extracellular hydrolytic enzymes produced by XooAS29. To obtain insight into the pathway by which THY oil exhibited these activities, we assessed the effect of oil on expression of genes significant for motility, EPS, lytic enzymes, virulence factors and DSF signaling in XooAS29 using RT-qPCR. The selected genes included the gum genes *gumC*, lytic enymes genes (xylanase, extracellular protease, endoglucanase, cellulase, 1,4-beta cellobiosidase, lytic enzyme, lipase/esterase, and polygalacturonase), DSF related biosynthetic genes *rpfF*, chemotaxis and motility genes *cheD, cheY, motA*, and *motB*, fimbrial protein gene *pilA*, flagellar genes *flgA* and *flgE*, and virulence gene *acvB*. The results showed that THY oil significantly down- regulated the expression of lytic enzymes genes such as extracellular protease (12%), lipase/esterase (12%), *acvB* (14%), chemotaxis and motility genes *motB* (19%), *cheD* (28%), *cheY* (11%), and *gumC* (18%), *rpfC* (38%), genes. Genes strongly suppressed were related to motility and flagellar biosynthesis genes such as *motA* (76%), flagellar gene *flgE* (60%), hydrolytic enzymes such as endoglucanase (75%), xylanase (64%), 1, 4-beta cellobiosidase (67%), and polygalacturonase (65%), along with DSF related gene *rpfF* (78%). Contrarily, the expression of cellulase, lytic enzyme, *flgA* and fimbrial protein *pilA* genes was not found to be significantly affected (**Figure 6**).

### THY Oil Restrains Synthesis of DSF and BDSF Using HPTLC

**FIGURE 7:**
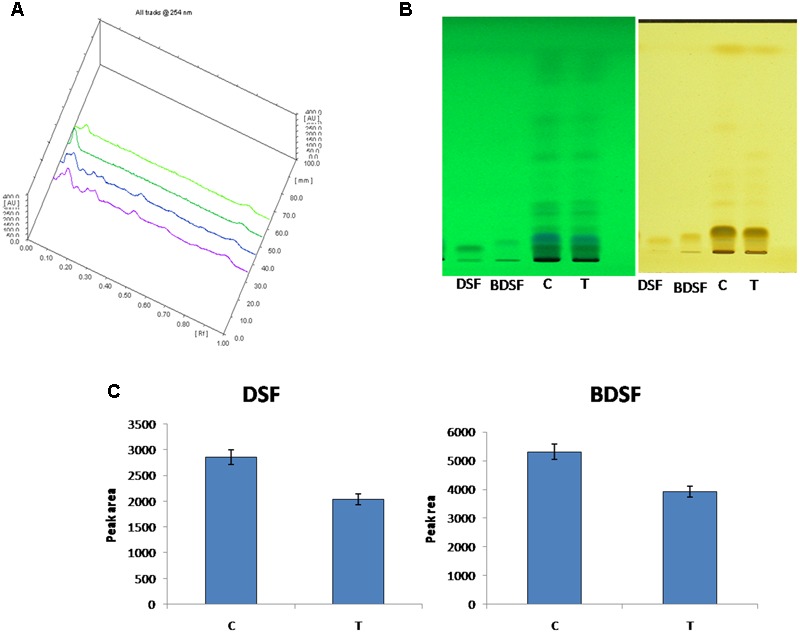
HPTLC analysis of DSF and BDSF levels in *X. oryzae* pv. oryzae (Xoo) strain AS29 in the presence and absence of 500 ppm THY oil. **(A)** Overlapping spectra; **(B)** UV at 254 and 366 nm and **(C)** Quantification of DSF and BDSF molecules. Control treatment having only Xoo cells is denoted by “C” while THY oil treated Xoo cells are refereed as “T”. Results are means of three technical replicates and three biological replicates and error bar indicates the standard error.

In Xoo, DSF and BDSF are key signaling molecules controlling virulence in response to cell density. The inhibitory effect of 500 ppm THY on production of both the molecules was also confirmed by HPTLC analysis (**Figure 7**). Separation of DSF and BDSF was attained using a mobile phase comprising of hexane–ethylether–acetic acid (8: 2: 1). Photography of the TLC plate was done at λmax 254 and 366 nm wavelengths in UV absorbance mode using the HPTLC system (CAMAG, Switzerland). The corresponding bands of DSF and BDSF were observed at Rf 5.9 and Rf 7.8, respectively.

### Virulence Assay

**FIGURE 8:**
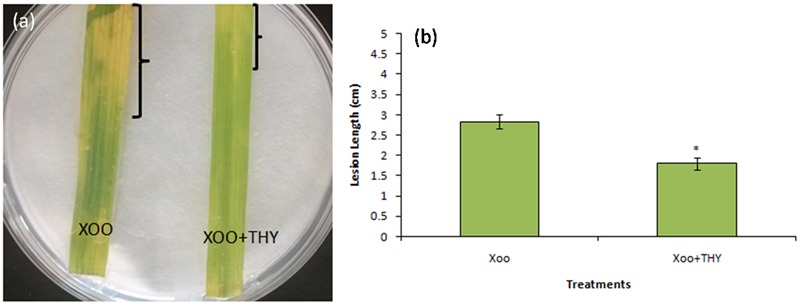
Thyme oil effects on rice disease responses to the representative XooAS29. **(a)** Leaves were inoculated with XooAS29 in the absence or presence of 500 ppm of THY oil using the leaf-clipping method. Only “Xoo” served as control while “Xoo+ THY” represented the treatment in the figure. Photographs were taken at 3 days after pathogen inoculation. (**b**) Lesion development was examined by measuring lesion length in XooAS29 in the absence or presence of 500 ppm of THY oil. Results are means of three technical replicates and three biological replicates and error bar indicates the standard error. Asterisk indicates ^∗^*P* < 0.05.

In order to gain insight into whether the decrement in the above mentioned genes and traits impaired XooAS29 virulence and disease inciting potential, the virulence assay by leaf clip method was conducted. The results showed that the effective concentration of 500 ppm THY oil enervated the virulence potential of XooAS29 in comparison to the control leaves infected with only Xoo 3 days after pathogen inoculation (**Figure 8**). Water agar plates having rice leaves revealed significant decrement in lesion length (1.57 fold) in THY oil treated rice leaves as compared to only Xoo treated control leaves.

### Gas Chromatographic Identification of the Individual Components of THY Oil

After THY oil was found to be potential in curtailing the pathogencity of XooAS29, our next step was to identify the individual components present in this oil. For this, THY oil was subjected to GC-FID apparatus. Thymol, gamma-terpinene, and para-cymene were found as major components of THY oil being 47.88, 29.61, and 20.15%, respectively. A representative image of the chromatogram is shown in Supplementary Figure S3.

### Rpf F Inhibition by Individual Components of THY Oil Confirmed by Molecular Docking

**FIGURE 9:**
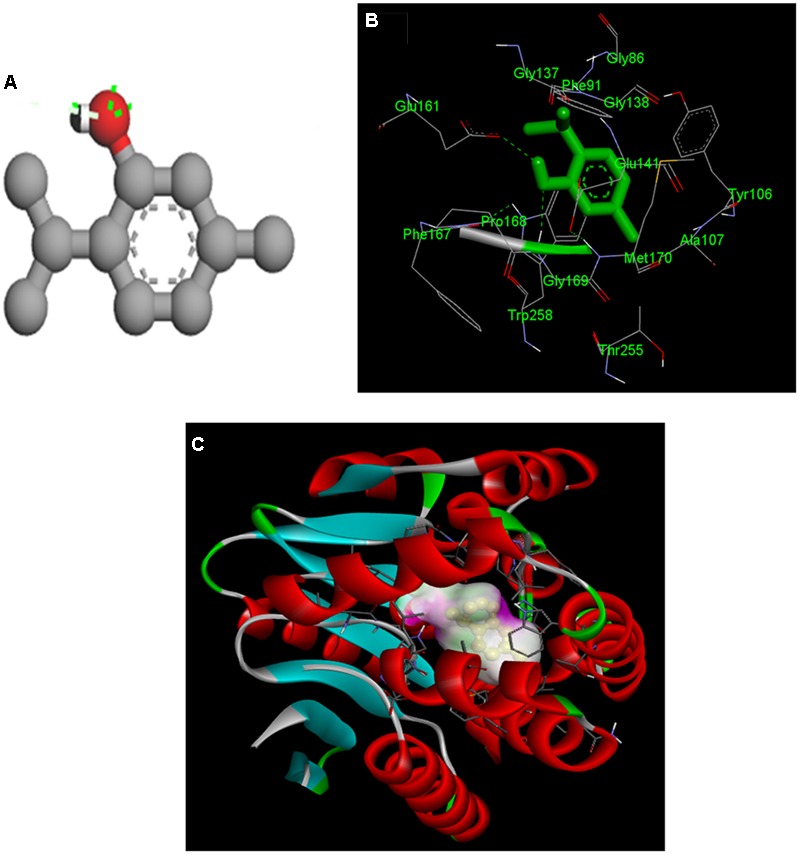
Molecular docking of the interaction between thymol and rpfF. **(A)** 3D structure of thymol. **(B)** Both thymol and ligand contact residues are represented and **(C)** binding orientation of thymol in RpfF. The protein is depicted as a ribbon, and secondary structures (i.e., helix, strand, and loop) are shown.

After identification of the individual components of THY oil as thymol, gamma-terpinene, and para-cymene, we expanded our finding toward to identification of the active component responsible for inhibition of signaling molecules using molecular docking. Silencing of the DSF synthase RpfF protein in *X. oryzae pv. oryzae*, could be a potential target protein to curb the infection. To study the possible inhibitory action of individual components of THY oil on DSF synthase RpfF protein, we used information available from the RpfF (PDB: 3M6M) to model the three-dimensional (3D) structure of the RpfF protein catalytic domain. Docking of thymol, gamma-terpinene, and para-cymene in the putative RpfF binding pocket indicated that these compounds formed potential hydrogen bonds with some residues of catalytic importance (**Figure 9** and Supplementary Figure S4). However, this structural model revealed a substantial region of interaction between thymol with RpfF protein, a positive regulator of DSF synthesis. The calculated binding energy of thymol, gamma-terpinene, and para-cymene was found to be -6.94, -5.99, and -5.79 kcal/mol, respectively.

### Effect of Thymol on XooAS29 Biofilm Formation, Growth Kinetics, and Selected Gene Expression

**FIGURE 10:**
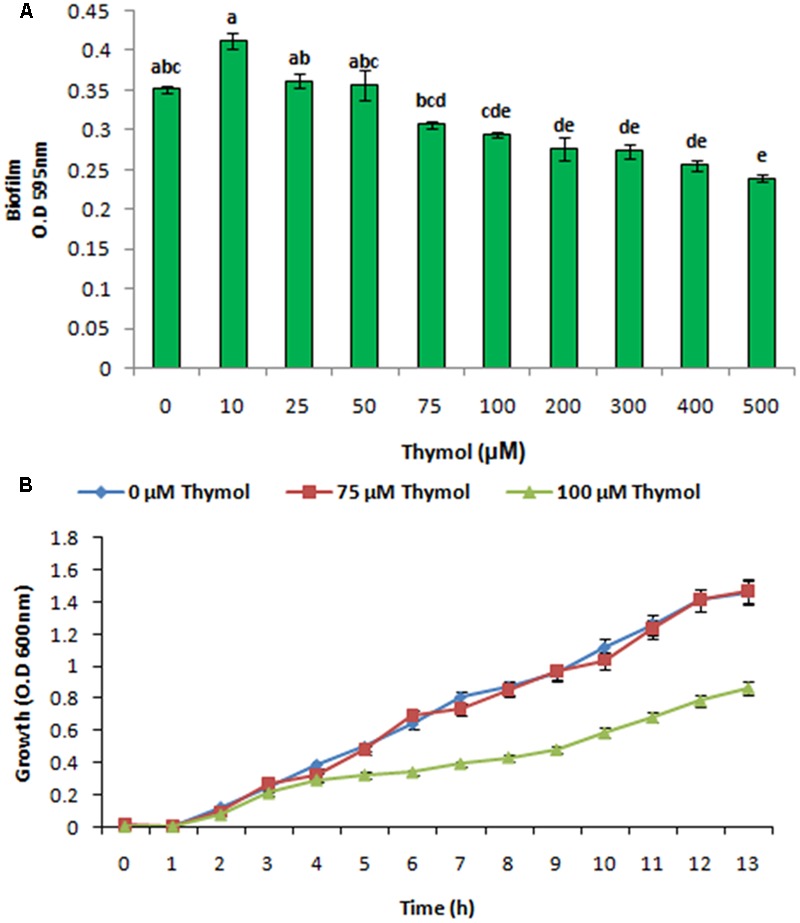
Effect of THY oil on *X. oryzae* pv. *oryzae* (Xoo) strain AS29 biofilm formation and colony forming units. **(A)** Biofilm formation and **(B)** growth kinetics at different concentrations of thymol (0, 10, 25, 50, 75, 100, 200, 300, 400, and 500 μM) for 24 h in microtiter plate. Results are means of three technical replicates and three biological replicates and error bar indicates the standard error. Different alphabets on vertical bars indicate significant dissimilarity among treatments (*P* < 0.01; Duncan’s multiple comparison test).

XooAS29 biofilm formation with thymol was evaluated using a static biofilm assay. Biofilm formation was reduced by thymol in a concentration dependent manner as shown in **Figure 10A**. The effect of thymol on XooAS29 growth was also evaluated by monitoring the OD of cells cultures at 600 nm (**Figure 10B**). The pattern of XooAS29 growth was not significantly different between the cultures with 0 and 75 μM thymol during the lag, exponential and stationary growth phases (**Figure 10B**).

**FIGURE 11:**
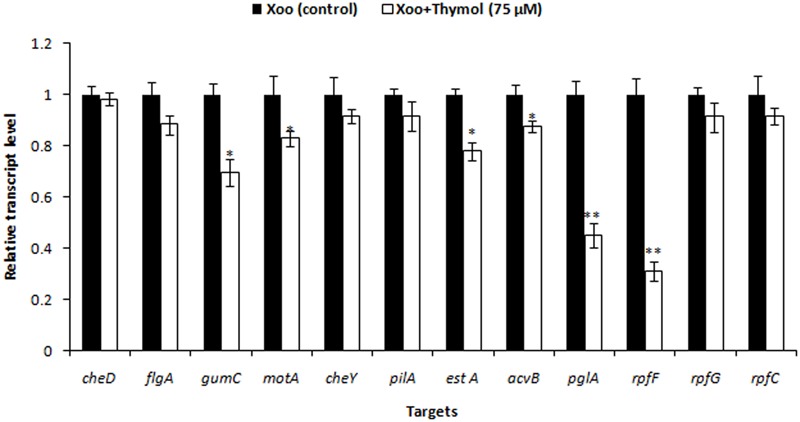
Differential gene expression in *X. oryzae* pv. *oryzae* (Xoo) strain AS29 in the absence and presence of Thymol (75 μM) as revealed by quantitative real time polymerase chain reaction (qRT-PCR) analysis. Only “Xoo” served as control while “Xoo+ THY” represented the treatment in the figure. *gyrB* was used as an endogenous control. Results are means of three technical replicates and three biological replicates and error bar indicates the standard error. Asterisk indicates ^∗∗^*P* < 0.01 and ^∗^*P* < 0.05.

Previously, we showed that 75 μM thymol inhibited XooAS29 biofilm formation without effective cell growth. As shown in supplementary data, the effect of thymol was evaluated on the expression of various genes responsible for motility, virulence factors and DSF signaling in XooAS29 using RT-qPCR. The results showed that thymol (75 μM) significantly down-regulated the expression of *gumC* (30%), *motA* (17%), *estA* (22%), virulence gene *acvB* (12%), and *pglA* (54%)along with *rpfF* (68%) while other genes such as *cheD, flgA, cheY*, and *pilA, rpfG*, and *rpfC* were not significantly affected by thymol (**Figure 11**).

## Discussion

Pathogenesis of many agriculturally important pathogenic bacteria is under the control of intercellular communication called QS. In QS based cell to cell conversation, bacteria senses change in density of its counterparts in reaction to extracellular signal molecules called autoinducers (AIs) and thereby modifies their gene expression. When a certain threshold level of AIs is accumulated, the bacteria set off a range of biological processes such as motility, hydrolytic enzymes and EPS biosynthesis (von Bodman et al., 2003; Singh et al., 2012). In the present investigation, we hypothesized that the aromatic oils used in traditional medicine system might contain QS blockers. Thus, effect of thyme essential oil isolated from the fresh leaves of *T. vulgaris* on biofilm formation potential and virulence factors produced by Xoo was examined. Biofilm formation and adherence to solid surface have been previously confederated with the virulence of many plant pathogenic bacteria like Xoo, *X. axonopodis* pv. *citri* (Xac) and others (Danhorn and Fuqua, 2007; Lim et al., 2008; Mccarthy et al., 2008; Gottig et al., 2009). However, despite of the significance of biofilm formation in bacterial pathogencity, strategies for developing an antimicrobial therapy are very poorly investigated especially in the field of agriculture. The results revealed that at 500 ppm, THY oil significantly reduced the biofilm formation which corroborated well with the reduced EPS and xanthomonadin secretion. Previous investigations have revealed the role of EPS in bacterial pathogenesis as loss of EPS was found to be positively related with loss of virulence (Sutton and Williams, 1970; Dharmapuri and Sonti, 1999). Likewise, Xanthomonadins apart from being the peculiar attribute of the genus safeguards the pathogen from its own defense mechanism, helps in epiphytic continuance and aids in host infection (Goel et al., 2002; He et al., 2011). In most of the bacteria, EPS production is an important deciding factor for formation of biofilm and hence, an inhibitor of motility (Dow et al., 2003; Jeong et al., 2008; Rosenberg et al., 2013). Interestingly, alleviation in swimming and swarming potential of XOOAS29 by THY oil may thus be possible by the antagonizing action of oil against QS mediated signaling. In a recent study conducted both swimming and EPS production in strain XKK12 were enhanced by exogenous application of DSF thus substantiating the significance of DSF signaling in the processes (Xu et al., 2015). Further, the observations from the gene expression experiment indicated THY oil to have a discrete mechanism responsible for reducing biofilm as expression of a number of biofilm formation related genes pertaining to chemotaxis and motility such as *motA, motB*, and *flgE* were significantly decreased. However, at this stage possibility of reduction by other mechanism cannot be ruled out. Interestingly, in a study two compounds D-leucine and 3-indolylacetonitrile (IAN) were found to prevent biofilm formation by *X. citri* subsp. *citri* on host leaves and various abiotic surfaces at a concentration lower than the minimum inhibitory concentration (MIC) (Li and Wang, 2014). Likewise, in another study exogenous addition of DSF restored EPS production in the Xoo rpfF knockout mutant indicating a definitive role of DSF in regulating EPS production in Xoo (Jeong et al., 2008; He et al., 2010).

The ability of Xoo to incite the disease depends on other pathways too for controlling the pathogenic web like type II secretion system (T2SS). This system apart from regulating the secretion of EPS also controls the activity of extracellular enzymes such as protease, pectinase, endoglucanase, polygalacturonate lyase, amylase, etc. (Wei et al., 2007; He et al., 2009). Reduced expression of endoglucanase, xylanase, cellobiosidase, and polygalacturonase in our investigation again points out the role of THY oil in targeting the virulence arsenal of Xoo. Similar results have been previously reported by Jeong et al. (2008) who showed reduced expression of genes associated with motility, EPS as well as exoenzymes in rpfB, rpfC, rpfF, and rpfG mutants. However, contradictory observations have been recorded by Chatterjee and Sonti (2002) who reported normal EPS and xylanase levels in *rpfF* mutant. The variability in observations recorded by various groups could possibly be the result of different culture media or Xoo strains used in the studies. In the present investigation, control of *rpf* genes by some unknown regulatory component cannot be ruled out as decrement in *rpfC g*ene was observed when thyme oil having different active components was applied to Xoo cells. However, *rpfC* gene expression remained insignificant when Xoo cells were treated with only thymol.

Since the results obtained highlighted the interruption of DSF signaling pathway, we further cross confirmed the results by analyzing the expression of genes involved in synthesis of DSF. The *rpfF* gene that controls the production of DSF, a signaling molecule regulating the synthesis of EPS and exoenzymes was found to be significantly reduced. Positive effect of *rpfF* was further verified as *X. axonopodis* pv. *glycines* rpfF mutant was observed to produce less CMCase, protease, endo-β-1,4-mannanase, polygalacturonate lyase and pectolytic activity than their wild-type counterparts (Thowthampitak et al., 2008).

HPTLC results further validated the gene expression results as reduced levels of DSF was observed in THY oil treated Xoo as compared to the untreated Xoo cells. In addition to change in the level of DSF QS signaling molecule, our results also indicated a role of THY oil in alleviating the level of BDSF which are reported to be accountable for EPS synthesis (He et al., 2010). Thus, decreased synthesis of EPS by THY oil may infer some part from its positive outcome from DSF and BDSF molecule synthesis. Overall since the results favored the hypothesis of reduction in virulence traits of XOOAS29 by application of THY oil, detached leaf assay for observing the lesion length caused by XOOAS29 in presence and absence of oil was conducted. The oil was found to significantly depreciate the lesion length probably by reducing the biofilm formation and other virulence traits expression. Similar observations were recorded by Li and Wang (2014) as reduced number of lesions was observed on leaves of grapefruit by foliar spray of D- leucine and IAN which impaired the infection causing capacity of *X. citri* subsp. *citri* by repressing biofilm formation.

**FIGURE 12:**
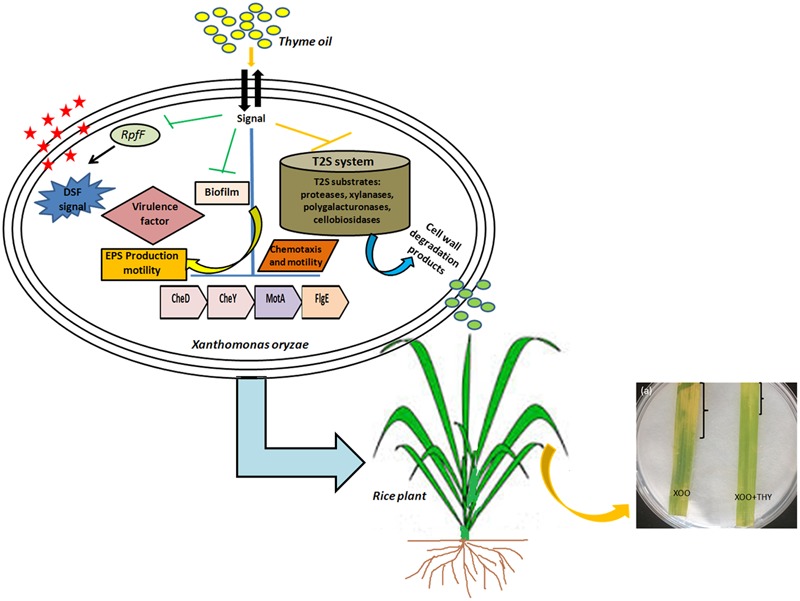
A schematic representation depicting alteration in QS pathway and virulence factors producing genes by thyme oil and its outcome on disease development in rice leaves.

Overall, the results suggest modulation of DSF QS circuits of XooAS29 which probably led to reduced biofilm formation, EPS, virulence and weakened infection (**Figure 12**). The results thus offer an attractive lead for the development of potent alternative for the development of anti-virulence alternative drugs for managing bacterial plant pathogens in agricultural fields. Further, bioinformatics study and wet laboratory experiments have revealed thymol to be a possible player as an anti-virulence agent since significant reduction in biofilm and genes pertaining to motility, chemotaxis, and virulence traits were observed. However, at this stage detailed gene level and knock out studies need to be done to identify the exact mode of action of both thyme oil and its active component thymol.

## Author Contributions

AS, RP: conceived and designed the experiments, manuscript writing. AS: performed and conceptualization of the experiments. RG: helped in Real time analysis. ST: GC analysis. RP: critical revision of the manuscript. All authors read and approved the final manuscript.

## Conflict of Interest Statement

The authors declare that the research was conducted in the absence of any commercial or financial relationships that could be construed as a potential conflict of interest.
