# Supplementary material for: Thyme Oil Reduces Biofilm Formation and Impairs Virulence of Xanthomonas oryzae
Source: Front Microbiol. 2017 Jun 13;8:1074. doi: 10.3389/fmicb.2017.01074 (PMC5468448; doi:10.3389/fmicb.2017.01074)
Supplement: Supplementary file 1 [file Data_Sheet_1.docx]

**Thyme Oil Reduces Biofilm Formation and Impairs Virulence of *Xanthomonas oryzae***

**Akanksha Singh^1^, Rupali Gupta^1^, Sudeep Tandon^2^ and Rakesh Pandey^1^***

*^1^Department of Microbial Technology and Nematology, CSIR- Central Institute of Medicinal and Aromatic Plants, P.O. CIMAP, Lucknow 226015, INDIA*

*^2^Chemical Processing Department, CSIR-Central Institute of Medicinal and Aromatic Plants, Lucknow 226 015, India*

***Mailing address:** E-mail: r.pandey@cimap.res.in; FAX: (+91) 522- 2719072;

Phone: (+91) 522 2718530

**Figure S1 Different concentrations of THY oil on biofilm formation and colony forming units of *X. oryzae* pv. oryzae (Xoo) strain AS29.** Results are means of three technical replicates and three biological replicates and error bar indicates the standard error. Different alphabets on vertical bars indicate significant dissimilarity among treatments (*P* < 0.01; Duncan’s multiple comparison test).

**
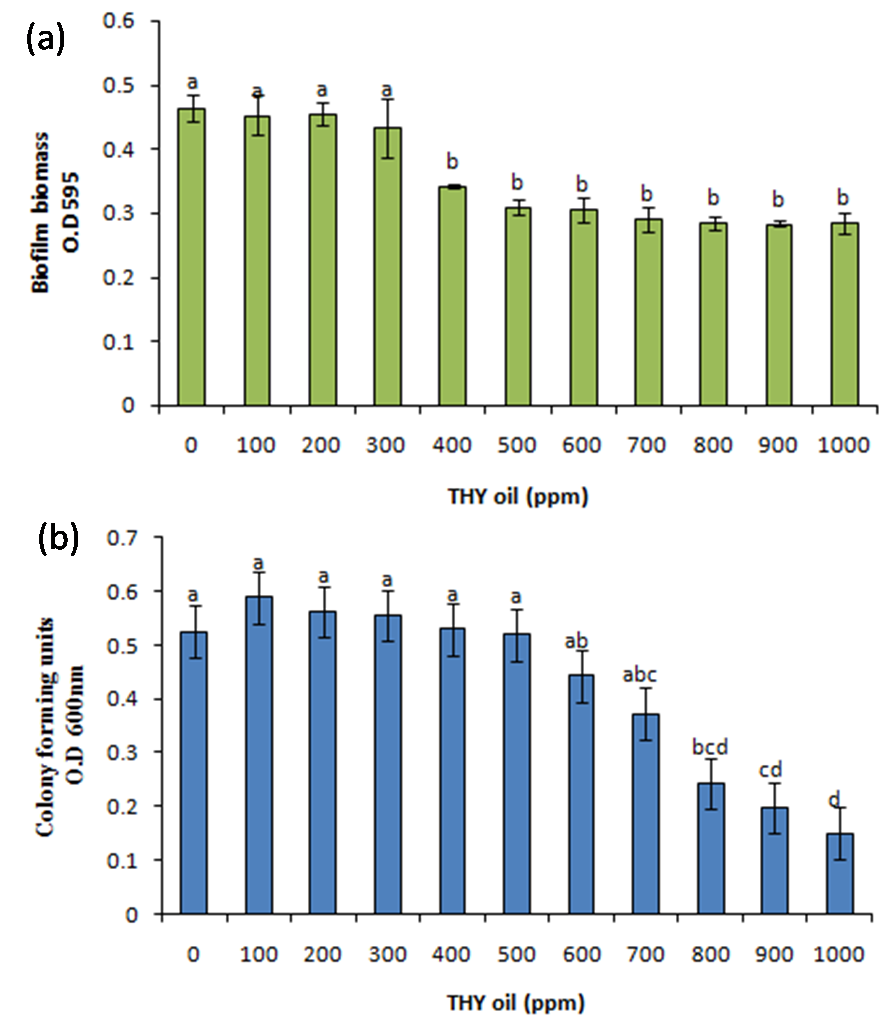
**

**Figure S2 Different concentrations of THY oil suppresses wetness of *X. oryzae* pv. oryzae (Xoo) strain AS29.** Results are means of three technical replicates and three biological replicates and error bar indicates the standard error. Asterisk indicates **P* < 0.05.


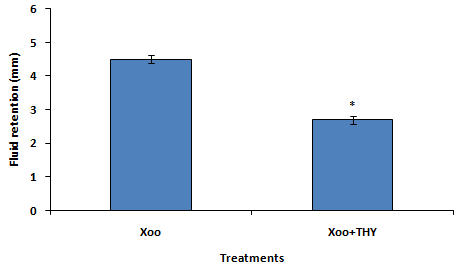


**Figure S3 GC-MS analysis of *Thymus vulgaris* essential oil (THY oil) for identification of individual components.**


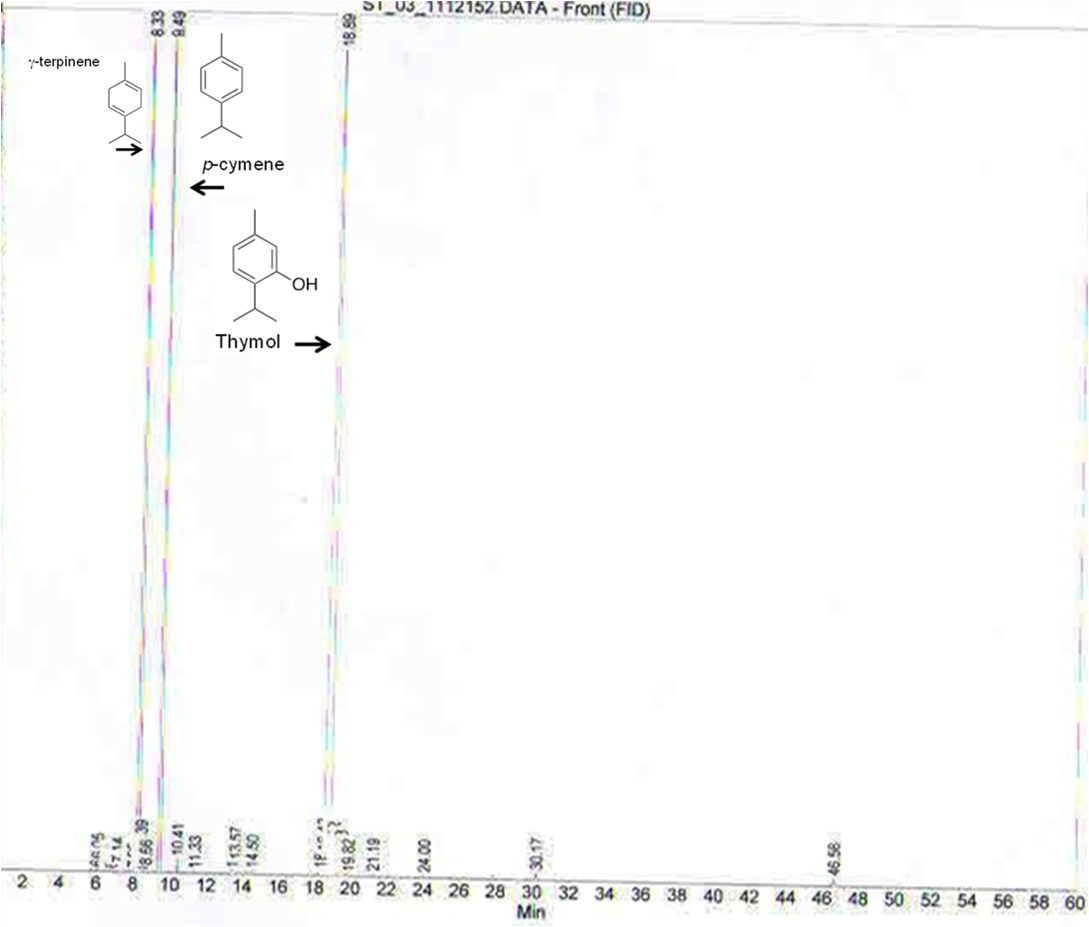


**Figure S4 Molecular docking of the interaction between gamma- terpinene, para -cymene and rpfF.** (A and D) 3D structure of gamma- terpinene and para –cymene respectively. (B and E) Both gamma- terpinene and para -cymene with ligand contact residues are represented respectively and (C and F) Binding orientation of gamma- terpinene and para -cymene in rpfF respectively. The protein is depicted as a ribbon, and secondary structures as helix, strand, and loop.
